# Point of Care Cardiac Ultrasound Applications in the Emergency Department and Intensive Care Unit - A Review

**DOI:** 10.2174/157340312801784952

**Published:** 2012-05

**Authors:** Robert T Arntfield, Scott J Millington

**Affiliations:** 1 Division of Critical Care and Division of Emergency Medicine, Western University, London, Ontario, Canada; 2 Department of Critical Care Medicine, The Ottawa Hospital / University of Ottawa, Ottawa, Ontario, Canada

**Keywords:** Echocardiography, emergency department, ICU, point of care, ultrasound.

## Abstract

The use of point of care echocardiography by non-cardiologist in acute care settings such as the emergency department (ED) or the intensive care unit (ICU) is very common. Unlike diagnostic echocardiography, the scope of such point of care exams is often restricted to address the clinical questions raised by the patient’s differential diagnosis or chief complaint in order to inform immediate management decisions. In this article, an overview of the most common applications of this focused echocardiography in the ED and ICU is provided. This includes but is not limited to the evaluation of patients experiencing hypotension, cardiac arrest, cardiac trauma, chest pain and patients after cardiac surgery.

## INTRODUCTION

Non-cardiologist use of ultrasound for rapid, bedside structural assessment of the heart in critically ill patients drew its earliest widespread attention in the early 1990s. It was shown that a rapidly performed, limited echocardiogram carried out by emergency physicians (EPs) could confer a mortality benefit to those with penetrating cardiac injuries [1] From this narrow scope, non-traditional users of echocardiography (echo) such as critical care physicians (CCPs) and EPs have expanded the applications of cardiac ultrasound to address a broad array of important clinical questions at the bedside. 

Point of care echo has expanded to the point where in the last two years, consensus statements from medical societies in both emergency medicine [2] and critical care [3] have been published, guiding practitioners on the appropriate scope of echo for these acute care providers. In considering the opposition to this "non-traditional" use of echocardiography, as recently as 10 years ago, [4] one can appreciate the significant progress that these documents signify. 

Though the emergency department (ED) and intensive care unit (ICU) represent distinct clinical environments, the indications and applications of basic echocardiographic assessment are quite similar. This commonality provides the backdrop for this review, which seeks to give the reader an understanding of the current "state of the art" application of this modality across a spectrum of critical illness, whether in the ED or the ICU. 

Different from diagnostic echocardiography, a limited or "focused" bedside echo is typically devoted to answering very specific clinical questions that are posed in response to a particular differential diagnosis, using the minimum and most efficient echo views and techniques. As such, this review is organized into sections based around common patient presentations, rather than specific diagnoses. Though the focus of the article is cardiac sonography, other applications of point of care ultrasound will be mentioned briefly in some sections. Readers interested in the broader scope of point of care ultrasound, as used in the ICU, ED and beyond, may enjoy the recent review by Moore and Copel [5].

## THE HYPOTENSIVE PATIENT

The identification and management of circulatory failure is a common occurrence in both the ED and the ICU. While traditional physical examination and hemodynamic monitoring (both invasive and non-invasive) often provide extremely valuable information, assessment of cardiac structures by ultrasound can rapidly provide data that would be otherwise inapparent. Items of particular interest to the clinician at the bedside include the assessment of left ventricle (LV) function, markers of the patient's volume status, and the presence or absence of pericardial effusion and/or tamponade physiology. 

### Diagnostic Accuracy in Hypotension

Not all causes of hypotension are amenable to echocardiographic diagnosis. Even under such circumstances, echo and ultrasound remain invaluable by rapidly paring down the long list of diagnostic possibilities. This contribution of ultrasound to medical decision making was shown in a randomized controlled trial where, in addition to usual care, an ultrasound protocol for patients with undifferentiated hypotension presenting to the ED was carried out. This protocol included, but was not limited to, assessment of the IVC, gross LV function and the pericardium. Taking an average of 6 minutes, the protocol lead clinicians to significantly narrow their differential diagnoses and increased overall diagnostic accuracy [6]. 

### LV Function

Assessment of LV function is one of the cornerstones of focused, point of care echo and, accordingly, will be mentioned throughout a variety of clinical scenarios in this article. With respect to undifferentiated hypotension, rapid assessment of global LV function is frequently carried out to determine whether any part of the patient's shocked state may be explained by poor left sided cardiac function. Determining that LV function is normal in patients with hypotension can be invaluable, and should expedite the consideration of non-cardiogenic causes for shock such as hypovolemia or sepsis. In such cases the clinician may be lead to prioritize support for the systemic vasculature with volume or vasopressors rather than supporting the heart itself with inotropic agents. In the alternative scenario (where LV function is found to be depressed in a patient who is in shock) enhancing cardiac output with inotropic agents may improve oxygen delivery once the patient is adequately fluid resuscitated [7] [8]. In either case, the primary focus must be to determine and treat the underlying cause of shock. The rapid, bedside determination of LV function to assist in diagnosis and management of patients in both the ED and the ICU has shown to be plausible: clinicians in both environments with minimal training in cardiac ultrasound have been shown to be capable of assessing LV function accurately, with excellent agreement with a blinded cardiologist's interpretation [9-11]. Further, when compared to invasively acquired data that have been frequently used to guide hemodynamic management decisions, a prospective single center trial showed good agreement between a CCP's echo interpretation of LV function and the cardiac index as determined by a pulmonary artery catheter (PAC) in a surgical intensive care unit [12].

### Volume Status

Identifying hypovolemia and/or a fluid responsive state in a hypotensive patient provides a clear, initial therapeutic path for the clinician. The simplicity of initiating aggressive fluid resuscitation is often tempered, however, by the difficulty in confidently determining a state of hypovolemia exists. Traditional methods of volume assessment such as jugular venous pressure, measurement of central venous pressure (CVP), or use of the PAC have each been scrutinized for their lack of accuracy in critically ill patients [13-15]. Conversely, it has long been known that echocardiographic assessment of the inferior vena cava (IVC) offers a compelling and accurate window in to the right sided filling pressures and overall volume status of the sick [16,17]. Over the past decade, physicians using ultrasound at the bedside have studied this application in acutely ill patients. IVC size and respiratory variation have been shown to correlate well with CVP (and its limitations) or volume responsiveness (the more relevant clinical parameter) in a variety of patient environments [11,18-21]. In addition to being technically easy to learn, IVC ultrasound is particularly well suited for the bedside provider (rather than the consulting cardiologist or echo technician) because frequent reassessment of IVC size after each intervention (such as the administration of fluids or inotropes) provides important feedback on the effect of these interventions and helps direct the next steps in management. 

More advanced echocardiographic techniques to assess volume status, including the use of continuous-wave Doppler to observe respiratory variation in volume-time integrals from the LV outflow tract [22] or the use of transesophageal echo (TEE) to assess superior vena cava size variation[23], have also been described with good results among advanced users in the ICU. Further, Tissue Doppler imaging is available on many portable ultrasound machines which, through assessment of myocardial tissue velocity during passive atrial filling, has shown promise in estimating volume status when studied by both EPs[24] and CCPs [25].

### Pericardial Effusion

With a broad list of medical causes, pericardial effusion is frequently considered in the differential diagnosis for patients who present with or develop hypotension or shortness of breath in the ED or ICU. Despite the elegant physiology that underlies physical exam findings such as the pulsus paradoxus or Beck's triad, their lack of sensitivity[26] has contributed to the evolution and adoption of point of care echo by clinicians seeking a more reliable diagnostic approach. The accuracy and safety of EP performed echo for has been well demonstrated in large prospective study of 515 patients where 103 pericardial effusions were detected and, using subsequent review by a cardiologist as the gold standard, produced a sensitivity of 96% and specificity of 98% [27]. The prevalence of effusion in high risk ED patients has been found to range from 13-20% [27,28]. In a smaller group of arrested or agonal patients, this prevalence was seen to rise to 40% [29]. The frequency with which pericardial effusions are found and their potential to cause significant hemodynamic embarrassment underscores the utility of limited echo in undifferentiated critically ill patients. 

Once a pericardial effusion is identified, determining its contribution to a patient's symptoms or hemodynamic instability (if present) is required. In addition to clinical data, the bedside clinician may seek out findings of either atrial or ventricular diastolic collapse to support a diagnosis of tamponade. In one prospective study of 110 patients with moderate to large pericardial effusions, the presence of right atrial or right ventricular diastolic collapse was noted in 90% of those with a clinical diagnosis of pericardial tamponade [30]. Of those patients without clinical tamponade, however, right chamber collapse was observed in 34%. Supporting the assertion that tamponade is foremost a clinical diagnosis,[31] these findings also show a notable lack of specificity to the "classic" 2D findings of diastolic collapse. As such, awareness of the potential limitations of these findings will help ensure that the clinical picture always takes priority over echocardiographic images in determining the immediate management of this problem. 

### The Septic Patient

Timely and aggressive management of severe sepsis and septic shock have become a focal point in ED and ICU culture for the past decade [7]. While the above descriptions of LV function and volume status assessments also hold true for septic patients, the variability in cardiac function seen in sepsis is worthy of its own brief discussion. Sepsis may have broad influences on the cardiovascular system, ranging from diffuse myocardial suppression to LV hyperdynamism. Identifying these findings on point of care echo may prove useful to the bedside clinician. 

For instance, in a cohort of hypotension ED patiets, hyperdynamic LV function was found to be highly specific for an eventual diagnosis of sepsis and thus has been raised as a potential aid to early diagnosis [32]. In the ICU environment, it has been shown in a series of 183 patients with septic shock that one third of patients suffer myocardial suppression related to their sepsis while the rest generally have hyperdynamic function [33]. These data underscore the value of echocardiography in hypotensive septic patients who, in addition to fluid resuscitation, ultimately may benefit from different forms of hemodynamic therapy – inotropes for those with myocardial suppression and vasopressors for those with preserved or hyperdynamic LV function. 

## THE PATIENT WITH CHEST PAIN

Atraumatic chest pain brings nearly 8 million people to visit EDs each year in the United States [34]. Immediate life threatening considerations amenable to assessment with echocardiography include myocardial ischemia, aortic dissection and pulmonary embolism. 

### Myocardial Ischemia

Typical ED investigations for chest pain thought to be due to coronary artery disease (CAD) include EKG and cardiac biomarkers. For high risk ED patients with negative investigations, many centers will admit for further risk stratification. Several studies support the use of both non-contrast and contrast echocardiography to risk stratify these patients at the bedside in the ED [35-37]. In particular, one study performed 2D echocardiography on 180 consecutive patients with chest pain being evaluated for CAD in the ED. EPs were blinded, and the echo findings were not used for clinical decision making. Of the forty-nine percent of patients who had no regional wall motion abnormality (RWMA), 98% went on to have a negative workup for CAD. Though assessment for RWMA is considered an advanced skill for EPs [3] this study raises the question of whether, with appropriate additional training, EPs could harness the negative predictive value of echocardiography to safely discharge their patients with chest pain. To this point, however, with no studies of EP-performed echo assessing for presence of absence of RWMA, echocardiographic assessment for CAD in the ED remains a component of formal diagnostic echo rather than part of any focused or limited bedside exam. 

### Aortic Dissection

**Fig. (2):**
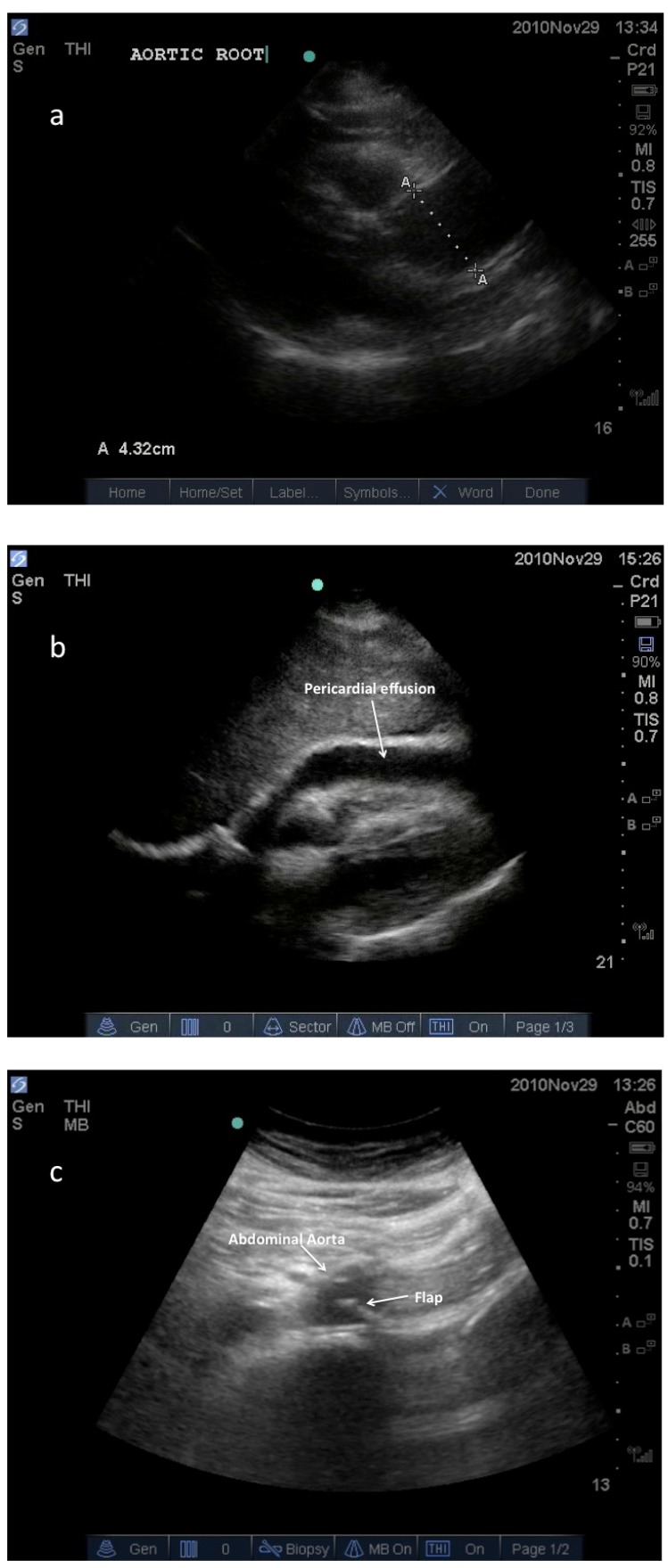
Images from a hemodynamically unstable patient who presented to the ED with undifferentiated chest pain and abdominal pain. Immediate point of care ultrasound was performed revealing the following images: **2a**: Parasternal long axis view demonstrating enlarged ascending aorta and aortic root. **2b**: Sub-xiphoid view demonstrating pericardial effusion. **2c**: Transabdominal view of the proximal abdominal aorta where a mobile, intraluminal, echogenic line was seen, suggestive of an intimal flap. After the patient was stabilized, CT scan confirmed a Stanford type A aortic dissection extending from the aortic root to the iliac bifurcation.

Aortic dissection is an uncommon but highly lethal condition that typically presents with chest pain and can be difficult to distinguish from acute myocardial infarction. In unstable patients where dissection is suspected, TEE is the diagnostic tool of choice, with sensitivity and specificity comparable to MRI or CT [38]. As TEE is infrequently performed by EPs, dissection is rarely diagnosed at the bedside by EPs except in rare circumstances using transthoracic echocardiography (TTE) (Fig. **2**) [39,40]. While the findings of a visible flap in the aorta (ascending or descending) or new aortic insufficiency on echo are supportive of the diagnosis, the absence of such findings are inadequate to exclude the diagnosis. Thus, if suspicion is high for aortic dissection, the physician's efforts will generally be better directed toward expediting a timely, definitive study such as CT scan, rather than devoting significant time to TTE. 

In the ICU environment, assessment for aortic dissection may be somewhat easier as TEE is a more commonly deployed tool. Due to technical challenges in excluding dissection (frequent ultrasound artifacts, blind spots, and false positives), however, TEE for this indication has been raised as a potentially perilous undertaking in non-expert hands [41]. Despite this, an international consensus statement includes detection of aortic dissection using TEE among a list of advanced competencies for CCPs [3]. The performance characteristics of TEE assessment for this diagnosis among non-cardiologists is, however, unknown. 

### Pulmonary Embolism

Pulmonary embolism (PE) is a challenging and resource consuming diagnosis for the EP. The pursuit of a bedside method to diagnose or exclude this condition is not new [42], but has been re-invigorated by the ability for clinicians to structurally assess the right heart on point of care echo. Findings such as right ventricular enlargement, new tricuspid regurgi-tation, and/or a hyperdynamic/underfilled LV occur in 27-55% of cases of PE [43,44]. Though these findings are associated with increased risk of death,[45] which may suggest an increased role for aggressive therapy such as thrombolysis,[46,47] the absence of such findings is insufficient to rule out PE entirely. Further, when findings of right heart strain on echo have been studied prospectively in the ED, a single center French study evaluating 76 hemodynamically stable patients determined to be at risk for PE (by high risk Wells score or elevated D-dimer), the echo findings carried a sensitivity of only 55% and specificity of 69% [48].

Thus, the role of echo in the ED for EPs caring for patients suspected of having PE of any size may rest with the triage of unstable patients for consideration of thrombolysis and is insufficient for ruling out the disease as a whole. 

The diagnosis of PE is often more troublesome in the ICU, particularly when considered as a cause of sudden circulatory failure (so-called "massive" PE). For these unstable patients, the hazard of transportation to the CT scanner in order to exclude PE is not insignificant. In a single center ICU-based study, a protocol of limited echocardiography and ultrasound of the deep venous system for thrombosis (DVT) in the assessment of 173 patients with known PE was undertaken [49]. It was found that 100% of patients with proximal (main, right or left pulmonary artery) embolism or hemodynamic instability had either signs of right heart strain (87%) or DVT (74%) on point of care ultrasound carried out by the clinician caring for the patient. While this study offers the promise of excluding hemodynamically significant PE at the bedside in ICU patients, replication of these findings in a multicenter fashion is required before this approach can be widely recommended. 

## THE PATIENT WITH CARDIAC TRAUMA

The rapid diagnosis of cardiac trauma, an injury which can be rapidly fatal whether penetrating or blunt, is an essential component of modern trauma care. 

### Penetrating Trauma

As referenced in the introduction of this article, the rapid evaluation for hemopericardium in patients suffering from penetrating thoracic trauma represents the earliest use of limited cardiac ultrasound by non-cardiologists. In a very simple retrospective review, Plummer *et al* evaluated the outcomes of patients with penetrating cardiac injuries before and after the implementation of EP performed echocardiography [1]. In 49 cases of penetrating cardiac injury, it was shown that those who received ED echo had a 100% survival, compared to 57.1% for those who did not. Given the time sensitivity of traumatic hemopericardium, this mortality difference was at least partly explained by the average of 27 minutes that separated the time to diagnosis between the 2 groups. Additional studies have demonstrated that a single view technique (typically sub-xiphoid) offers nearly perfect performance for detecting and ruling out hemopericardium across a number of different providers, including EPs, trauma surgeons and their trainees [50-53]. While this test performs with near perfect sensitivity, providers must be aware of pitfalls, including false positive exams caused by epicardial fat [54] and the uncommon but important false negative exam that may be caused by communication between a lacerated pericardium and a neighboring pleural space, thus creating hemothorax in lieu of hemopericardium [55].

### Blunt Trauma

The role for focused echocardiography in the detection of blunt cardiac trauma is less well defined. Although there are a few case reports related to contained cardiac rupture after blunt trauma,[56-58] the pericardial assessment that usually accompanies the assessment for intraperitoneal fluid in most trauma ultrasound ("FAST") exams is often non-contributory. Subtle findings, such as RWMAs suggestive of myocardial contusion or valvular incompetence as a marker of blunt valve injury are well beyond the scope of the trauma ultrasound exam of the heart. Similarly, assessment for blunt aortic injury is best carried out by expert TEE exam or computed tomography [59].

## THE PATIENT IN CARDIAC ARREST

Focused echocardiography in cardiac arrest can rapidly provide the clinician with both diagnostic and prognostic information. Originally studied by the cardiology community [60], the increasing presence of ultrasound machines in both EDs and ICUs engendered great interest among physicians for the application of echo in their patients with cardiac arrest of unknown etiology. With respect to prognosis in particular, there have been several compelling and widely quoted articles which have demonstrated that the absence of cardiac motion at any stage of the resuscitative effort suggests no opportunity for return of spontaneous circulation [61-63].

Despite calls for the incorporation of cardiac ultrasound into the ubiquitous ACLS algorithms [64,65], there is a paucity of evidence to support that cardiac ultrasound can improve patient outcome, which lead the most recent international guidelines on advanced life support to conclude "there is insufficient evidence to support or refute the routine use of ultrasound or echocardiography to guide cardiac arrest resuscitation" [66]. With a multi-center trial studying the use of TTE in cardiac arrest patients in the ED underway, additional data will likely be available to inform future iterations of this guideline. 

This same document appropriately raises the concern that routine use of TTE for patients in cardiac arrest may contribute to unacceptable interruptions in cardiopulmonary resuscitation (CPR). While the relationship between CPR and TTE requires further study, the use of TEE during cardiac arrest would obviate this concern and has shown feasibility in a case small series carried out in the ED [67]. Employing TEE as a live, 2D hemodynamic monitor during cardiac arrest, while posing technical challenges in an ED or ICU, may be considered analogous to the now routine use of TEE to guide the re-initiation of cardiac activity and weaning from cardiopulmonary bypass during cardiac surgery [68] and may represent an area of future study for clinicians. 

## THE MECHANICALLY VENTILATED PATIENT

The patient undergoing mechanical ventilation offers unique echocardiographic considerations for the clinician. While the majority of mechanically ventilated patients can be satisfactorily imaged via a transthoracic approach, significant failure rates have been described in a variety of ICU-based studies including failure rates as high as 85% in those having undergone cardiac surgery [69-71]. This has contributed to the growing clinical interest regarding use of TEE by CCPs with some European centers having embraced this approach for nearly 20 years [72], as well as the inclusion of TEE as an advanced competency for CCPs seeking competence in critical care ultrasound [3]. In addition to providing reliable images for some of the structural assessments described earlier, TEE has offered a new understanding of the way the heart's function is influenced by positive pressure ventilation [73]. 

In addressing unexplained hypoxemic respiratory failure, echocardiography remains the gold standard to exclude right-to-left shunts [74]. Though outside the scope of this article, sonographic assessment of respiratory failure in most ICUs will also include thoracic ultrasound. The use of ultrasound to directly assess the lungs in this setting includes not only the detection of pleural fluid but also the diagnosis of pneumothorax [75,76], pulmonary edema [77,78], and pulmonary consolidation [79].

Functional hemodynamic monitoring, the application of a therapy (for example a fluid bolus) to diagnose a particular physiologic state (intravascular hypovolemia, for example), is a means to facilitate the achievement of core critical care goals, including the optimization of stroke volume, afterload, or contractile state (to name only a few). The belief that improved patient outcomes will result from optimizing these parameters early in the course of critical illness is widely held, yet difficult to prove [80]. Echocardiography is an ideally suited tool to assess progress towards the achievement of these goals. The classic and best studied example of functional hemodynamic monitoring relies on heart-lung interactions (ventilation-induced changes in physiologic variables) to assess preload responsiveness (increased cardiac output in response to a fluid bolus). Assessment can be done by TEE or TTE, in spontaneously breathing or mechanically ventilated patients. The variation of specific parameters (such as vena cava diameter or LV stroke volume) may be measured in response to various stimuli such as volume infusion or changes in intrathoracic volume due to respiratory variation, yielding valuable information as to the state of preload responsiveness [81,82]. In particular, it has been shown that a 36% or greater change in superior vena cava diameter on TEE with tidal breathing accurately predicts an increased cardiac output in response to a fluid bolus [83].

With regards to myocardial contractility, echocardiography provides a reliable tool to assess changes in global and regional LV function in response to therapeutic maneuvers such as inotropic or volume therapy [84]. Since both LV preload and afterload are influenced by intrathoracic pressure, TEE may also permit the optimization of ventilator variables such as positive end-expiratory pressure (PEEP) [85,86] or the use of recruitment maneuvers [87] by direct visualization of their effects on cardiac size and function. 

Finally, echocardiography may be of use in the population of mechanically ventilated patients who cannot be separated from the ventilator. Myocardial ischemia may occur in response to increased respiratory workload, resulting in the failure of a spontaneous breathing trial. Although detection of myocardial ischemia is, as previously discussed, usually beyond the scope of point of care echocardiography, it has been demonstrated that this phenomenon can be readily detected using TEE-derived estimates of LV filling pressures [88].

## THE POST-CARDIAC SURGERY PATIENT

In many centers, CCPs are responsible for the care of critically ill post operative patients, including following cardiac surgery. While the use of echocardiography during cardiac surgery has become extremely prevalent and increasingly endorsed [89], its role in the immediate post-operative period has been more difficult to define. Although post-operative TEE has been shown to frequently yield valuable information [90], often resulting in adjustments to medical therapy [91], a clear understanding of when and why patients should undergo TEE in the immediate post-operative period remains elusive. TTE, on the other hand, is used relatively infrequently in this patient population due to frequent difficulties in image generation [92] and an inability to reliably visualize the pericardium [93]. The strongest argument in favour of an increased role for TEE may be its reassuring safety profile - morbidity related to probe insertion has been estimated between 0.1 and 1.2% with related mortality approaching zero [94-96].

The detection of pericardial tamponade is the classic indication for TEE in the post-cardiac surgery patient who is deteriorating. Given the poor performance of other diagnostic modalities such as chest x-ray and clinical examination [97,98], echocardiography remains the gold standard for diagnosis [99]. However, extreme care must be exercised in attempting to diagnose this cryptic phenomenon by TEE, as post-operative effusions are almost universally small and localised, and often fail to show the classic features of tamponade physiology [93].

Information regarding ventricular function is frequently useful in a post-cardiac surgery patient who is evolving poorly. TEE allows accurate determination of left-ventricular function to be made using a wide range of techniques both quantitative and qualitative, preload dependent and independent [100]. Segmental wall motion analysis performs better than ECG for the detection of post-operative myocardial ischemia [101], although assessment of RWMA remains a technique reserved for advanced echo practicioners. Newer techniques such as strain imaging offer the potential of a more accurate determination of regional and global LV function [102,103], but are technically difficult to perform and remain outside the scope of practice for the vast majority of CCPs. 

The difficulties in assessing of right ventricular function [104], even under optimal conditions, are only magnified after cardiac surgery. While classic 2D techniques are fraught with inaccuracies and pitfalls, 3D imaging may offer a much more accurate evaluation. Indeed the near universal deterioration seen on post-operative 2D imaging of the RV may be due to changes in geometry rather than function [105], a pitfall which may be more readily avoided using 3D techniques. Despite these theoretical advantages, 3D echo presents significant challenges with respect to equipment cost and operator expertise, and its application to critically ill patients has not been studied. 3D imaging is unlikely to enter mainstream use by CCPs in the near future, remaining a tool applied by cardiologists in very specific circumstances. 

Finally, TEE offers a relatively safe way to investigate an assortment of other common post-operative problems such as pleural effusions [106], diastolic dysfunction [107] localization of the source of embolic phenomenon [108], and the troubleshooting of misplaced pulmonary artery catheters [109]. 

## ADVANCED APPLICATIONS IN THE ICU

The declining use of pulmonary artery catheters in many institutions has motivated a search for less invasive means to estimate cardiac filling pressures in order to guide resuscitative efforts, and for general hemodynamic monitoring. Tissue Doppler imaging, a technique for measuring the velocity of myocardial tissue, is a key component in the assessment of diastolic function in echocardiography laboratories [110,111]. Although traditionally measured using a transthoracic approach, the accuracy of TEE-derived estimation of filling pressures in critically ill patients has been studied, and performs well when compared to “gold standard” invasive measures [112,113]. Since standard 2D assessment of the IVC is an effective method of gauging fluid status in the ED and the ICU, the advantages of these advanced methods must be weighed against the additional training and experience required to master them. 

An accurate, non-invasive measurement of cardiac output (CO) is often considered to be the holy grail of hemodynamic monitoring, and can be estimated both by TTE and TEE [114,115]. Although the quantification of CO has never been shown to improve patient outcome, it is nonetheless used in many institutions to aid in the titration of fluids, inotropes and vasopressors. Estimation of CO by echo is done using the hydraulic orifice formula, multiplying the cross-sectional area of any valve by the flow velocity across it [116]. A transthoracic approach is appealing due to its non-invasiveness and the ease with which parallel alignment with the LV and RV outflow tracts can be achieved, while the transesophageal technique offers improved accuracy in the measurement of cross-sectional area, a key source of error in CO calculation. 

While ultrasound has gained widespread recognition for its ability to improve safety during central venous catheter insertion [117,118], the use of ultrasound to guide pericardiocentesis has also been extensively studied [119-121]. In one particular study, involving 110 consecutive patients undergoing echo-guided pericardiocentesis, only 1 death was directly ascribed to the procedure, with an overall complication rate of 20% (with most complications being relatively minor) [114]. As EPs are frequently screening for the presence of pericardial fluid in hemodynamically unstable patients, they are occasionally in the position of having to perform emergent pericardiocentesis. The use of ultrasound may offer improved safety and comfort in these circumstances. 

The concept of ruling out severe valvular regurgitation or stenosis in an unstable patient as part of an ICU or ED screening echocardiography protocol has been proposed [122,123], and recognition of massive valvular regurgitation has been recommended as a basic component of screening echocardiography [3]. However, valvular assessment is fraught with pitfalls and remains challenging even for very experienced echocardiographers [124], suggesting that non-cardiologists should exercise caution in the interpretation of valvular findings in this patient population. Similarly, quickly excluding the possibility of infective endocarditis or other endovascular infection in acutely septic patients seems clinically important, and indeed diagnostic echocardiography remains the centerpiece of this process. Again, caution is warranted as there are technical challenges posed by imaging cardiac masses [125] in addition to a lack of studies supporting the echocardiographic diagnosis of this condition by non-cardiologists. 

## CONCLUSIONS

From modest beginnings just over 20 years ago, focused point of care echocardiography performed by non-cardiologists has become an accepted component of the practice of emergency and critical care medicine. With its incorporation into post-graduate training programs worldwide, echo is being rightfully acknowledged as fundamental part of an acute care clinician's assessment of a critically ill patient. Key factors favoring the proliferation of focused echocardiography include cheaper and smaller ultrasound machines, prolific research demonstrating safety and feasibility, political leadership from various medical societies, and the vision and teaching of collaborative cardiologists. 

As reviewed in this article, there is already a broad array of echo applications available to physicians to enhance their care of critically ill patients, with many new applications awaiting further research. As training of non-cardiologists evolves and becomes increasingly standardized, the use of focused bedside echocardiography can be expected to grow dramatically. As such, in addition to applying new and exciting indications for bedside echo, a priority for all clinicians must continue to be the safe and responsible oversight of its existing applications. 

## Figures and Tables

**Fig. (1):**
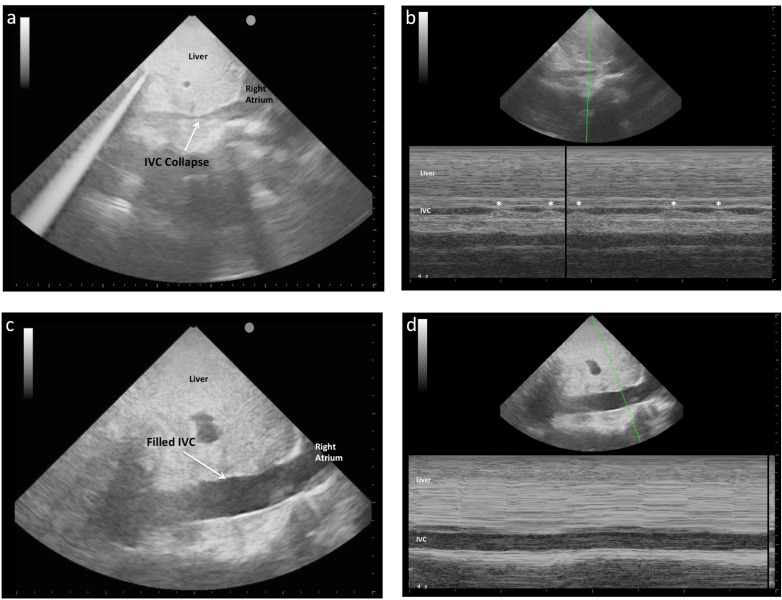
IVC images taken in the same spontaneously breathing patient with sepsis at the bedside in the ED. **1a** 2D image shows a small calibre IVC collapsing during spontaneous breathing. **1b** M-mode image of the same patient shows collapse with each breath (denoted by '*'). **1c** 2D image of the same patient after 90 minutes and 3L of crystalloid shows larger calibre IVC and, as is seen in 1d An M-mode demonstrates the absence of size variation with respiration, suggestive of adequate fluid resuscitation.

**Fig. (3):**
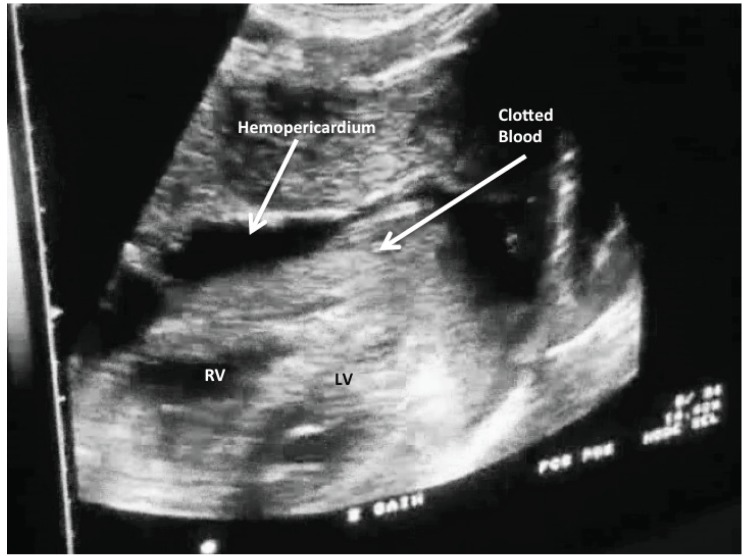
Hemopericardium as seen from a sub-xiphoid view after a penetrating injury to right ventricle (RV). Note clotted blood in pericardium.

**Fig. (4):**
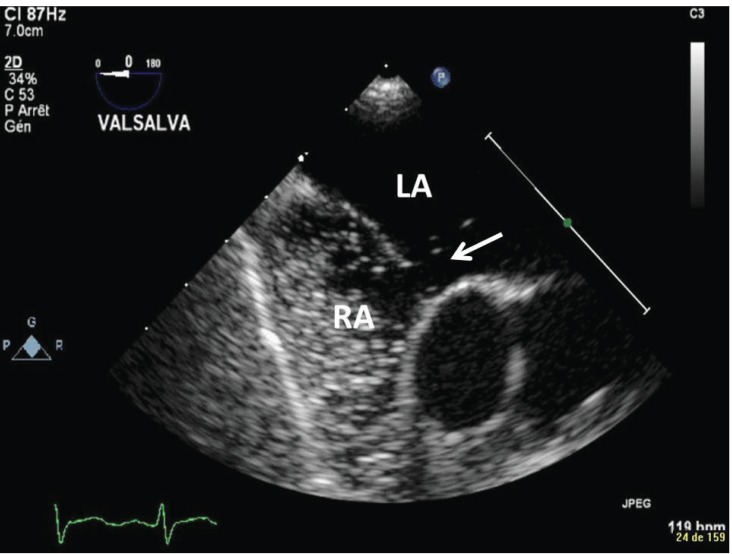
Right-to-left shunt. On this modified mid-esophageal bicaval view from a bedside, focused TEE in the ICU, agitated saline bubbles (arrow) can be seen passing from the Right Atrium (RA) to the Left Atrium (LA) through a defect in the intra-atrial septum.

## ABBREVIATIONS

ED: = Emergency department
ICU: = Intensive care unit
EP: = Emergency physician
CCP: = Critical care physician
LV: = Left ventricle
CVP: = Central venous pressure
IVC: = Inferior vena cava
PAC: = Pulmonary artery catheter
TEE: = Transesophageal echocardiography
CAD: = Coronary artery disease
RWMA: = Regional wall motion abnormality
TTE: = Transthoracic echocardiography
PE: = Pulmonary embolism
DVT: = Deep venous thrombosis
RV: = Right ventricle
CPR: = Cardiopulmonary resuscitation
PEEP: = Peak end expiratory pressure
CO: = Cardiac output
